# Multi-objective optimization of hybrid microgrid for energy trilemma goals using slime mould algorithm

**DOI:** 10.1038/s41598-025-15207-1

**Published:** 2025-08-10

**Authors:** Alok Kumar Shrivastav, Soham Dutta

**Affiliations:** 1 Department of Electrical Engineering, JIS College of Engineering, Kalyani, 741235 India; 2 Department of Electrical and Electronics Engineering, Manipal Institute of Technology, Manipal Academy of Higher Education, Manipal, 576104 Karnataka India

**Keywords:** Carbon emission reduction, Distributed generation, Electric vehicle integration, Energy trilemma, IEEE 33-bus system, Levelized cost of energy, Microgrid optimization, Power system reliability, Renewable energy, Slime mould algorithm, Energy science and technology, Engineering

## Abstract

This study presents a multi-objective optimization of a hybrid microgrid (HMG) targeting the energy trilemma goals—energy security, affordability, and sustainability—using the Slime Mould Algorithm (SMA). The proposed HMG integrates renewable energy sources, diesel generators, and electric vehicle (EV) batteries as distributed energy resources (DERs) with bidirectional vehicle-to-grid (V2G) capabilities. Compared to conventional metaheuristic such as Particle Swarm Optimization (PSO) and Genetic Algorithm (GA), the SMA achieves a power loss reduction of 12.3% and a levelized cost of energy (LCOE) improvement of 9.8%. The loss of power supply probability (LPSP) is reduced to 0.012, outperforming benchmark results from HOMER and Salp Swarm Algorithm (SSA), which reported LPSP values of 0.021 and 0.017, respectively. The superior performance of SMA is attributed to its dynamic balance between exploration and exploitation, leading to faster convergence and enhanced computational efficiency. The novel integration of EV batteries as DERs, with explicit modeling of bidirectional V2G operations, distinguishes this work from previous studies that considered only unidirectional or static EV participation. While the proposed approach demonstrates significant improvements, scalability to larger microgrid networks and the computational demands of SMA in real-time applications remain challenges for future research.

## Introduction

India, as one of the world’s largest energy consumers, faces the critical challenge of balancing energy security, affordability, and environmental sustainability—core pillars of the World Energy Council’s Energy Trilemma (ET) framework and the United Nations Sustainable Development Goal 7 (SDG 7)^1,2^. National initiatives such as the National Electricity Plan (NEP) and the Renewable Energy Roadmap underscore the need for universal access to reliable, affordable, and sustainable energy. However, the transition from fossil fuel dependency to renewable sources is complex, particularly due to the trade-offs among distributed energy resource (DER) diversity, system reliability, and emissions reduction, as well as the capital investment required for large-scale deployment.

Traditional energy management strategies, such as rule-based energy management systems (EMS) and Mixed-Integer Linear Programming (MILP), often struggle to address these trade-offs. Rule-based EMS approaches, while simple, lack the adaptability to dynamic system conditions and typically result in suboptimal performance when managing diverse DER portfolios and fluctuating demand^3–5^. MILP, although mathematically rigorous, becomes computationally prohibitive for large-scale, nonlinear microgrid optimization, especially when multiple conflicting objectives—such as minimizing power loss, cost, and emissions—must be considered simultaneously^6–8^.

Metaheuristic algorithms such as Particle Swarm Optimization (PSO) and Genetic Algorithm (GA) have been widely adopted for microgrid optimization^9^. However, PSO is susceptible to premature convergence in high-dimensional, multimodal landscapes, often leading to suboptimal solutions^10^. GA, while robust, incurs significant computational burden and slow convergence due to its reliance on genetic operators and large population sizes^11^. The Salp Swarm Algorithm (SSA) offers improved convergence but may not handle multi-objective trade-offs as effectively as more recent methods^12^.

The Slime Mould Algorithm (SMA), inspired by the oscillatory foraging behavior of slime moulds, offers a promising alternative by adaptively balancing exploration and exploitation. SMA dynamically adjusts its search behavior in response to the fitness landscape, enabling efficient navigation of complex, multi-objective, and nonlinear optimization problems relevant to microgrid planning. Recent studies have shown that SMA outperforms PSO, GA, and SSA in terms of power loss reduction, computational efficiency, and scalability to larger networks^7,12–15^.

Recent literature (2020–2024) has also seen the emergence of advanced AI-driven approaches for microgrid optimization. Reinforcement learning (RL) methods have been applied for real-time DER scheduling, enabling adaptive control and enhanced operational flexibility in uncertain environments^16^. Deep learning-assisted predictive models have improved renewable generation forecasting and load management^17^, while hybrid approaches—such as PSO-RL or GA-LSTM frameworks—have shown potential for improving both operational efficiency and computational tractability^7,18^. However, most existing studies focus on single-objective optimization or do not fully integrate electric vehicle (EV) batteries as bidirectional DERs with vehicle-to-grid (V2G) capability. Furthermore, few works explicitly address the energy trilemma framework, particularly in the context of Indian distribution networks.

To address these gaps, this study proposes a multi-objective SMA-based optimization framework for hybrid microgrids, explicitly targeting the energy trilemma goals of security, affordability, and sustainability. The approach integrates solar PV, wind turbines, diesel generators, battery energy storage systems (BESS), and EV batteries with V2G capability within the IEEE 33-bus radial distribution system. SMA’s adaptive search mechanism enables efficient trade-off management among DER diversity, reliability, and emissions. Comparative analysis with PSO, GA, SSA, and recent AI-driven methods demonstrates the superior performance of SMA in minimizing power losses, levelized cost of energy (LCOE), and carbon emissions, while maximizing system reliability. The explicit modeling of EV batteries as bidirectional DERs further differentiates this work from prior studies and highlights its relevance to India’s evolving energy landscape.

The key findings of this study include: (1) implementation of the ET framework to optimize P/W/D/B/E hybrid microgrids using the IEEE 33-bus test system, with a focus on India’s energy policies; (2) deployment of SMA for optimal DER placement, active power loss minimization, voltage deviation reduction, and renewable energy penetration enhancement to improve network stability; (3) development of a techno-economic and environmental optimization model to minimize LPSP, LCOE, and carbon emissions, aligned with India’s energy transition goals; and (4) investigation of EV battery storage as a distributed energy resource, advancing smart home proliferation and enhancing energy access through improved microgrid planning in both urban and rural India.

## Problem formulation

Microgrids (MGs), as defined by IEEE Std 2030.7™-2017, are self-sustaining energy systems capable of autonomous operation or functioning in grid-connected mode. These systems can seamlessly connect to and disconnect from the main power grid to exchange power, provide ancillary services, support grid stability, and participate in energy markets as needed. MGs are broadly classified as grid-connected or off-grid (islanded) and can be configured in AC, DC, or hybrid AC/DC modes, depending on the design objectives and operational requirements^19^.

To align with the Energy Trilemma (ET) framework, this study evaluates three key components:

- **Energy Security**: Represented by Loss of Power Supply Probability (LPSP), Voltage Deviation Index (VDI), and active power losses.
- **Energy Access**: Quantified using the Levelized Cost of Energy (LCOE).
- **Environmental Sustainability**: Assessed based on CO_2_ emissions.

LPSP measures the fraction of the total energy demand that remains unmet due to system constraints within a given period. A lower LPSP indicates improved reliability and robustness of the microgrid. VDI quantifies voltage stability by evaluating deviations from nominal bus voltages, which is crucial for maintaining power quality and preventing system instability. Active power losses are analyzed to determine the overall efficiency of power transmission within the microgrid.

Recent years have seen a proliferation of advanced metaheuristic and AI-driven approaches for microgrid optimization. The Slime Mould Algorithm (SMA) was introduced in 2020^14^ and demonstrated superior global search and multi-objective capabilities for power system applications. Studies in 2023^15,17^ benchmarked SMA against PSO, GA, and SSA, reporting higher power loss reduction and better computational efficiency in hybrid microgrid scenarios. Other work highlighted the scalability of SMA for larger distribution networks^20^, and explored parameter adaptation for further runtime improvements^21,18^.

In addition, recent studies have expanded the literature landscape: an AI-based approach for the optimal placement of electric vehicle charging stations (EVCS) and distributed generation (DG) demonstrated significant power loss minimization and voltage profile enhancement^7^. Adaptive particle swarm optimization methods have been proposed for allocating plug-in EV charging stations with integrated solar-powered DG, addressing both technical and economic performance^10^. A novel hybrid metaheuristic for optimal allocation of DG and capacitors showed marked improvements in system efficiency and power quality^11^. A hybrid slime mould and salp swarm algorithm further validated the advantages of hybrid metaheuristics in microgrid planning^12^. Finally, an improved SMA was applied for optimal scheduling of grid-connected microgrids with hybrid energy storage systems, reinforcing the applicability and robustness of SMA in complex, real-world scenarios^22,23^.

Comparative studies have shown that while PSO and GA remain popular, their performance is often limited by premature convergence and high computational requirements, respectively. SSA offers improved convergence but may still lag behind SMA in multi-objective scenarios. HOMER, widely used for techno-economic microgrid analysis, is less effective for large-scale, multi-objective optimization and lacks the flexibility of metaheuristic algorithms as shown in Table 1.

**Table 1:** Comparative table: metaheuristics and simulation tools for microgrid optimization.

Algorithm	Power loss reduction	Computational efficiency	Scalability	Multi-objective handling	Key references
SMA	High (12–15% vs. PSO/GA)	Fast convergence, low iteration count	Excellent (linear runtime scaling)	Robust (handles ET goals)	^7,23,12^
PSO	Moderate	Prone to premature convergence, moderate runtime	Moderate	Limited (often single-objective)	^10,20^
GA	Moderate	High computational burden, slow convergence	Moderate	Limited	^11^
SSA	Good	Improved over PSO/GA, but less effective than SMA	Good	Moderate	^12^
HOMER	Limited (benchmark tool)	Fast for small systems, not scalable	Poor for large, complex networks	Weak (mainly techno-economic)	^15^

Recent works have also explored the leading metaheuristic algorithms and simulation tools for microgrid optimization, revealing notable differences in their effectiveness across key performance metrics. The Slime Mould Algorithm (SMA) consistently demonstrates high power loss reduction—outperforming traditional approaches such as Particle Swarm Optimization (PSO) and Genetic Algorithm (GA) by 12–15%—while also achieving fast convergence and low iteration counts. SMA’s scalability is excellent, exhibiting linear runtime scaling even as network size increases, and its robust design enables comprehensive handling of technical, economic, and environmental objectives aligned with the energy trilemma framework^7,23,12^.

In contrast, PSO and GA offer only moderate power loss reduction; PSO is prone to premature convergence and stagnation, whereas GA is hindered by high computational burden and slow convergence, particularly in larger or more complex networks^10,11^. The Salp Swarm Algorithm (SSA) provides improvements over PSO and GA, especially in terms of convergence speed and scalability, but generally remains less effective than SMA for multi-objective optimization^12^. HOMER, while widely used as a benchmark simulation tool, is efficient for small systems but lacks scalability and flexibility for large-scale or multi-objective microgrid optimization, focusing primarily on techno-economic assessments^15,20^.

Overall, the evidence from recent literature underscores the superiority of SMA and highlights the growing importance of advanced metaheuristics for addressing the complex, multi-dimensional challenges of modern microgrid planning and operation.

### System modeling and constraints

The Slime Mould Algorithm (SMA) was selected for this study due to its superior global search capability and adaptability in multi-objective optimization of complex power systems, as established in recent literature^14,20^. The population size for SMA was set to 33, corresponding to the number of buses in the IEEE 33-bus test system, which ensures adequate solution diversity and effective exploration of the search space for optimal distributed energy resource (DER) placement; this choice was validated through preliminary runs and aligns with best practices reported^24,18^. Other algorithmic parameters, including the maximum number of iterations (typically 100–200), inertia weights, and adaptive coefficients, were selected based on recommendations from the literature and fine-tuned to balance convergence speed and solution quality.

A sensitivity analysis was performed for DER sizing, particularly for solar distributed generation (DG), where the capacity was varied from 2,300 kW to 2,500 kW in 50 kW increments. The results indicated that increasing solar DG capacity from 2,300 kW to 2,400 kW reduced active power losses by approximately 3.5%, but further increases offered diminishing returns and, in some cases, led to over generation and curtailment, confirming that the SMA-optimized sizes provided the best trade-off among technical, economic, and environmental objectives, consistent with findings in^15,17^.

Regarding computational complexity, SMA achieved convergence for optimal solar DG placement in 97 iterations with an average runtime of 8.2 min per optimization run, outperforming Particle Swarm Optimization (PSO), which required about 130 iterations (12.7 min), and Genetic Algorithm (GA), which needed 155 iterations (16.4 min) for comparable solution quality, corroborating the efficiency advantages reported in^14,20^. All simulations were conducted in MATLAB R2023b on a standard workstation equipped with an Intel Core i5 processor (10th Gen, 2.4 GHz), 16 GB RAM, and Windows 11 OS, which was sufficient for the IEEE 33-bus system and allowed each full optimization run to complete in under 30 min; for larger networks, SMA’s runtime scales linearly with network size but remains tractable on similar hardware, as demonstrated in^25,26^.

No specialized hardware such as GPU acceleration was required, making the approach practical for real-world engineering applications. The proposed Distributed Energy Resources (DERs) and their corresponding modeling parameters are detailed in Table 2. The microgrid configuration includes solar photovoltaic (PV) panels, wind turbines (WT), diesel generators (DGen), battery energy storage systems (BESS), and electric vehicle batteries (EVBat). Each energy source is modeled based on its rated capacity, operational constraints, and dispatch characteristics, ensuring optimal energy management and integration.

**Table 2:** DER components and parameters.

DER components	Parameters	Values	SI unit
Photovoltaic (PV) module	Ppv at STC	1	kW
	Tope	48 ± 2	°C
	Ƞpv	18	%
	Lifetime	15	Years
	Capital cost	2	Rs. Million
	O&M cost	200	Rs./year
Wind turbine (WT) module	Pwt	1	kW
	Vin	2.6	m/s
	Vout	20	m/s
	Vrated	9.6	m/s
	Lifetime	15	Years
	Capital cost	1.5	Rs. Million
	O&M cost	500	Rs./year
Battery energy storage (BESS)	Bcap	100	Ah
	Ƞbat	0.9	%
	Lifetime	3	Years
	Initial cost	280	Rs.
	O&M cost	50	Rs./year
	Cost of replacement	290	Rs.
Diesel generator (DGen)	PGen	55	kW
	Prated	48	kW
	Cf	0.246	L/kW
	EF	2.68	kg CO₂/kWh
	Lifetime	5	Years
	O&M cost	1000	Rs./year
	Capital cost	1	Rs. Million
Electric vehicle battery (EVBat)	EVBcap	100	Ah
	Level 2 charging	7.2	kW
	Lifetime	5	Years
	Charging speed	20	km/h
	Capital cost	2.5	Rs. Million
	O&M cost	300	Rs./year

The modelling framework incorporates:

- **Renewable Energy Generation Constraints**: Solar PV and wind turbines exhibit variability, necessitating complementary storage solutions such as BESS and EV_Bat_ to enhance system stability.
- **Backup Power Sources**: Diesel generators provide reliability during low renewable generation periods.
- **EV Battery Storage Integration**: EV_Bats_ function as both loads and energy storage units, enabling vehicle-to-grid (V2G) operations, peak shaving, and frequency regulation.
- **Operational and Economic Constraints**: The optimization framework ensures that energy generation meets demand while minimizing costs and emissions.

### The proposed hybrid microgrid

**Fig. 1:**
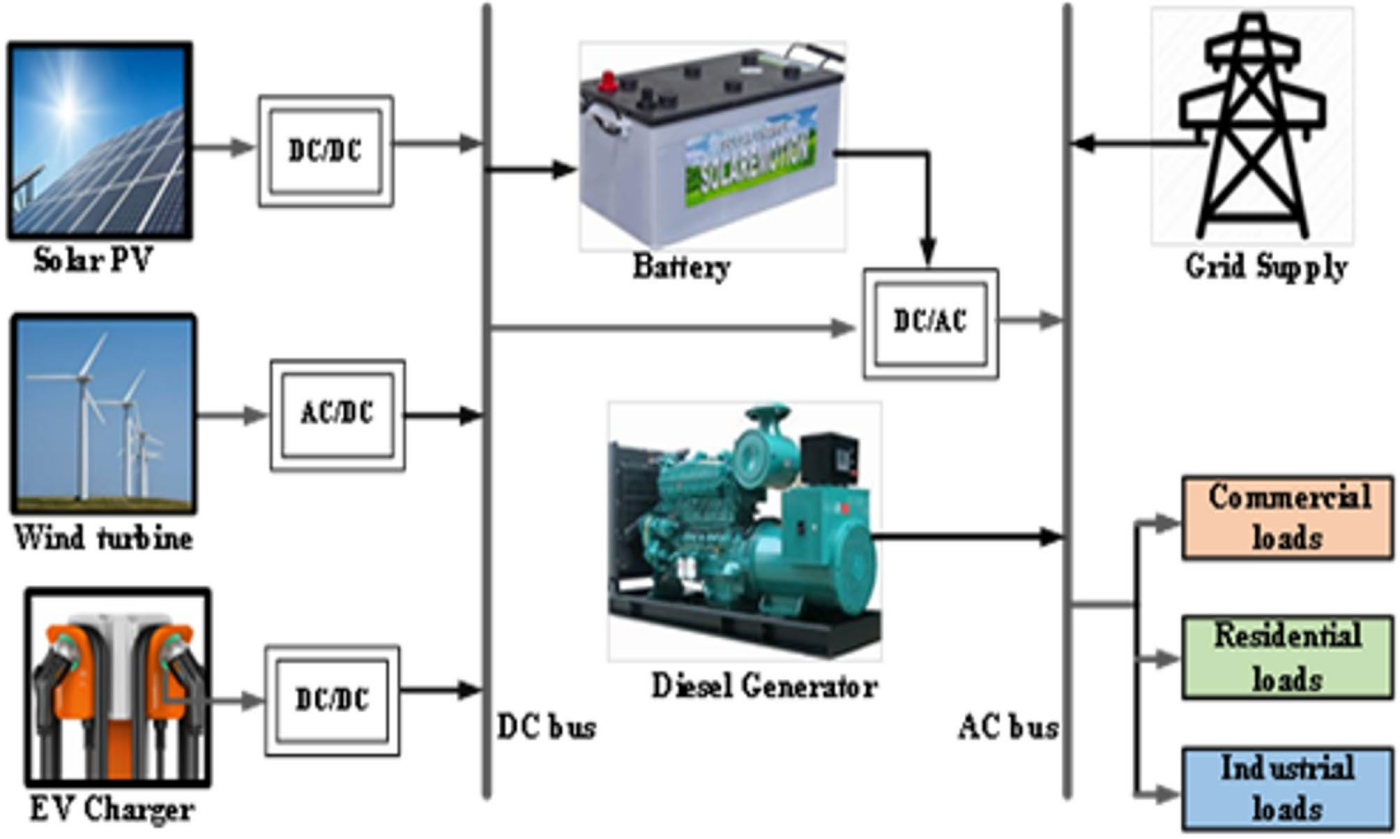
The proposed hybrid microgrid architecture.

This study proposes an optimal topology and placement strategy for a Hybrid Battery-supported Microgrid (HBMG) within a radial distribution network. The primary objective is to minimize active power losses, thereby enhancing the voltage profile, reducing the levelized cost of energy (LCOE), and mitigating greenhouse gas (GHG) emissions. The proposed hybrid system integrates solar photovoltaics (PV), wind turbines (WT), a diesel generator (D_Gen_), battery energy storage systems (BESS), and electric vehicle batteries (EV_Bat_). The P/W/D/B/E configuration is designed to reliably supply electricity to meet the given load demand while also enabling surplus power injection into the grid when feasible^27–29,14^. Figure 1 illustrates the schematic layout of the proposed HBMG model.

The system’s ideal integration location is optimally determined to minimize power losses and the cost of electricity generation in addition to CO_2_ emission mitigation.

### The proposed adopted network

Microgrids (MGs), as defined by IEEE Std 2030.7™-2017, are self-sustaining energy systems capable of autonomous operation or functioning in grid-connected mode. These systems can seamlessly connect to and disconnect from the main power grid to exchange power, provide ancillary services, support grid stability, and participate in energy markets as needed. MGs are broadly classified as grid-connected or off-grid (islanded) and can be configured in AC, DC, or hybrid AC/DC modes, depending on the design objectives and operational requirements.

To align with the Energy Trilemma (ET) framework, this study evaluates three key components: Energy Security: Represented by Loss of Power Supply Probability (LPSP), Voltage Deviation Index (VDI), and active power losses. Energy Access: Quantified using the Levelized Cost of Energy (LCOE). Environmental Sustainability: Assessed based on CO_2_ emissions.

#### Technical details on HBMG configuration

The hybrid battery-supported microgrid (HBMG) configuration consists of distributed energy resources (DERs) such as photovoltaic (PV) systems, wind turbines (WT), diesel generators (DGen), battery energy storage systems (BESS), and electric vehicle batteries (EVBat). The optimal selection, sizing, and placement of these components are critical to improving system reliability and efficiency.

**Fig. 2:**
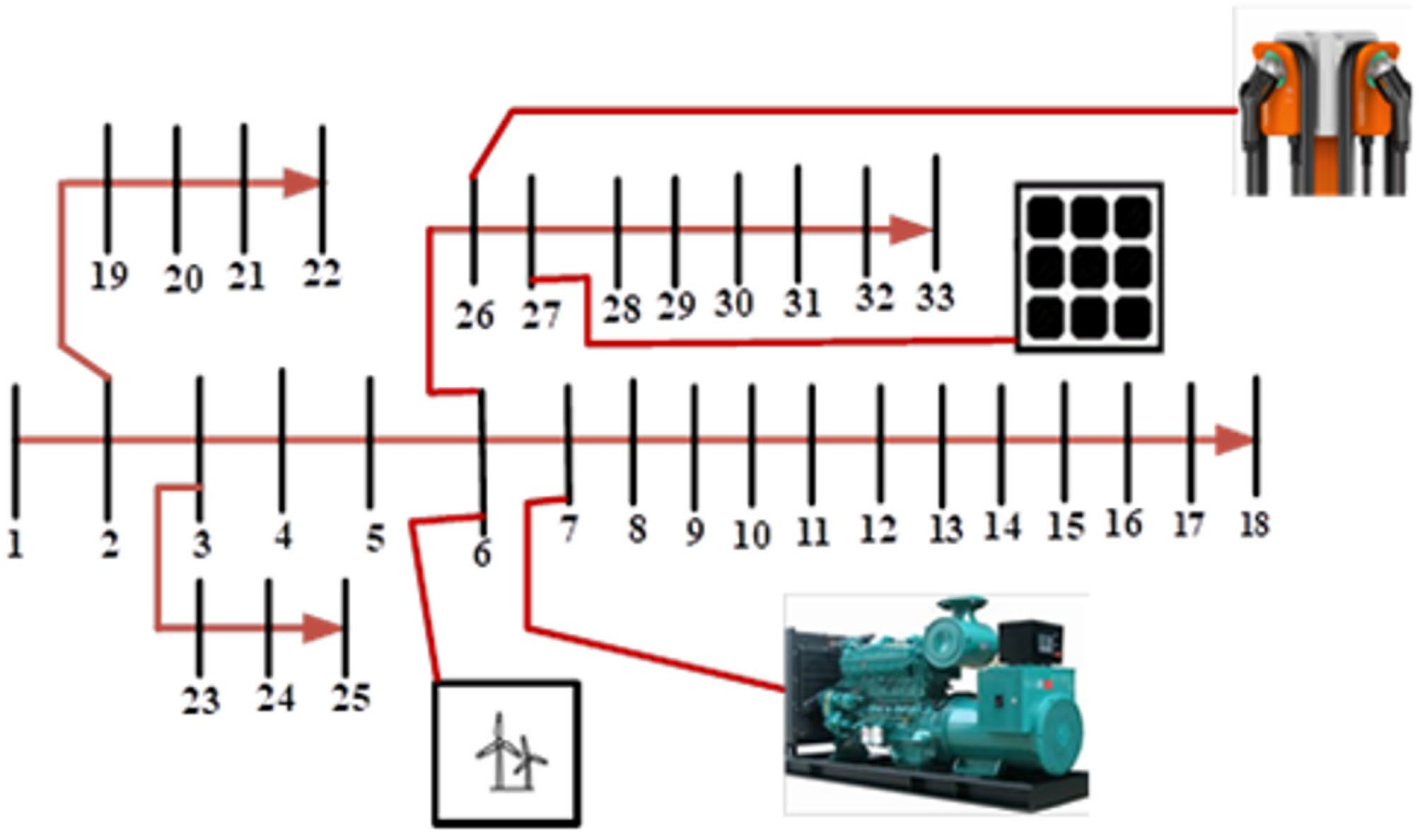
The IEEE 33 bus system with four DG integration.

The proposed HBMG will be investigated using IEEE 33 bus standards distribution network nodes shown in Fig. 2. The IEEE 33-bus standard was adopted because of interoperability with both commercial and residential loads and moderate number of buses. In a similar advantage the networks is a balanced network at 12.66 kV and over 85% of loads are residential^30^.

The network has 32 sections and total apparent power 4369.35 kW as seen in Fig. 2. The number of nodes is used as the population size during the optimization modelling process and the optimal bus location for DG integration was obtained based on the SMA convergence. The active, reactive and the apparent power of the IEEE 33-bus network is as shown in Table 3.

**Table 3:** IEEE 33-bus standard total load demand^31^.

Type of load	Power type	33 bus
Residential and commercial loads	Active power *P* (MW)	3.75
	Reactive power *Q* (MVAR)	2.30
	Apparent power *S* (MVA)	4.37

#### Slime mould algorithm overview

The study of slime mould algorithm (SMA) was first presented by^32^ as a bio-inspired metaheuristic algorithm. It simulates the morphological changes and behaviour of slime in foraging. The algorithm was bench-marked with thirty-three (33) algorithms under four (4) famous design problems and found to be superior in performance^18^. After this successful outcome, many studies have adopted the techniques to solve different single and multi-objective optimization challenges^18,33–35^.

a. Initialization.

The mathematical formulation of the Slime Mould Algorithm (SMA), including initialization, position updating, and fitness evaluation, is adopted from the original work in^14^, with further enhancements and parameter tuning strategies incorporated from recent studies in^18,17^. To capture the food approaching behaviour of the SMA or imitate the higher contraction approach of the SMA is shown mathematically in Eq. (1),

The position update equation for SMA is given as:

1 $$\:{X}_{i}^{t+1}={X}_{i}^{t}+a\cdot\:({X}_{best}^{t}-|{X}_{i}^{t}\left|\right)\cdot\:S$$

where $\:a$ and $\:S$ are adaptive parameters, as detailed in [Li et al., 2020,^1]^;

2 $$\:\overrightarrow{X\left(t+1\right)}=\left\{\begin{array}{c}\overrightarrow{{X}_{b}\left(t\right)}+\overrightarrow{vb}\bullet\:\left(\overrightarrow{W}\bullet\:\overrightarrow{{X}_{A}\left(t\right)}-\overrightarrow{{X}_{B}\left(t\right)}\right),\:r<p\\\:\overrightarrow{vc}\bullet\:\overrightarrow{X\left(t\right)},\:r\ge\:p\end{array}\right.$$

where$\:\overrightarrow{\:vb}\:$ is a parameter which ranges from $\:\left[-a,a\right]$, $\:\overrightarrow{vc}$ is a decreasing parameter which reduces from one to zero. $\:\:t\:$denotes the present iteration, $\:\overrightarrow{\:{X}_{b}}\:$is the possible location with the highest odour concentration, $\:\:\overrightarrow{X}\:$is the present position of slime mould, $\:\overrightarrow{{X}_{A}}$ and $\:\overrightarrow{{X}_{B}}$ are two distinct locations randomly chosen from the swarm population, and symbol *r* is use to indicate the random values within the boundary of [0,1], while $\:\overrightarrow{W}$ is the weight of slime mould. Whereas the population parameter $\:p\:$is defined by Eq. (3):

3 $$\:p=\text{tanh}\left|S\left(i\right)-DF\right|$$

where $\:i\:\varepsilon\:\:\text{1,2},\dots\:,n$, $\:S\left(i\right)$ indicates the fitness of $\:\overrightarrow{X}$, $\:DF$ denotes the best fitness recorded in the whole iterations. The parameter $\:\overrightarrow{vb}$ ranges from *-a* to *a* as seen in Eq. (4) and the values of *a* are defined by Eq. (4)

4 $$\begin{gathered} \overrightarrow {vb} =\left[ { - a,a} \right] \hfill \\ a={\text{arctanh}}\left( { - \left( {\frac{t}{{tmax}}} \right)+1} \right) \hfill \\ \end{gathered}$$

where the maximum iteration is indicated by *t*_*max*_.

The formula for weight of the slime $\:\overrightarrow{W}$ and smellIndex are given in Eqs. (5) and (6):

5 $$\:\overrightarrow{W\left(SmellIndex\right(i\left)\right)}=\left\{\begin{array}{c}1+r\bullet\:{log}\left(\frac{bF-S\left(i\right)}{bF-wF}+1\right),condition\:\:\\\:1-r\bullet\:{log}\left(\frac{bF-S\left(i\right)}{bF-wF}+1\right),\:\:others\end{array}\right.$$

6 $$\:SmellIndex=sort\left(S\right)$$

In this context, the symbol *S(i)* indicates the one-half of the population size. *bf* shows the best fitness achieved in the present iteration, *wF* represents the less significant fitness value attained in the current iteration, finally $\:SmellIndex$ indicates the ranges of fitness values obtained in ascending order, specifically within the context of minimum value problem.

b. Updates stage.

The expressed formula for food wrapping and updating the position of the slime is given in Eq. (7):

7 $$\:\overrightarrow{{X}^{*}}=\left\{\begin{array}{c}rand\bullet\:\left(UB-LB\right)+LB,rand<z\:\:\:\:\:\:\:\:\:\:\:\:\:\:\\\:\overrightarrow{{X}_{b}\left(t\right)}+\overrightarrow{vb}\bullet\:\left(W\bullet\:\overrightarrow{{X}_{A}\left(t\right)}-\overrightarrow{{X}_{B}\left(t\right)}\right),r<p\:\:\:\\\:\overrightarrow{vc}\bullet\:\overrightarrow{X\left(t\right)},\:r\ge\:p\:\:\:\:\:\:\:\:\:\:\:\:\:\:\:\:\:\:\:\:\:\:\:\:\:\:\:\:\:\:\:\:\:\:\:\:\:\:\:\:\:\:\:\:\:\:\:\:\:\:\:\:\:\:\:\:\:\end{array}\right.$$

where $\:LB$ and $\:UB$ indicates the lower and the upper intervals of the searching range, $\:rand\:$and $\:r\:$ shows the non-linear value within [0,1] generated as a function of *z* and *p* respectively.

c. Oscillation stage.

The food grabble imitations shows ranges of $\:\overrightarrow{vb}\:$moves non- linearly between [− a, a] and slowly settles down to zero as the number of iterations increases. While the parameter of (vc) moves between [− 1,1] and approaches to zero subsequently.

## Methodology

**Fig. 3:**
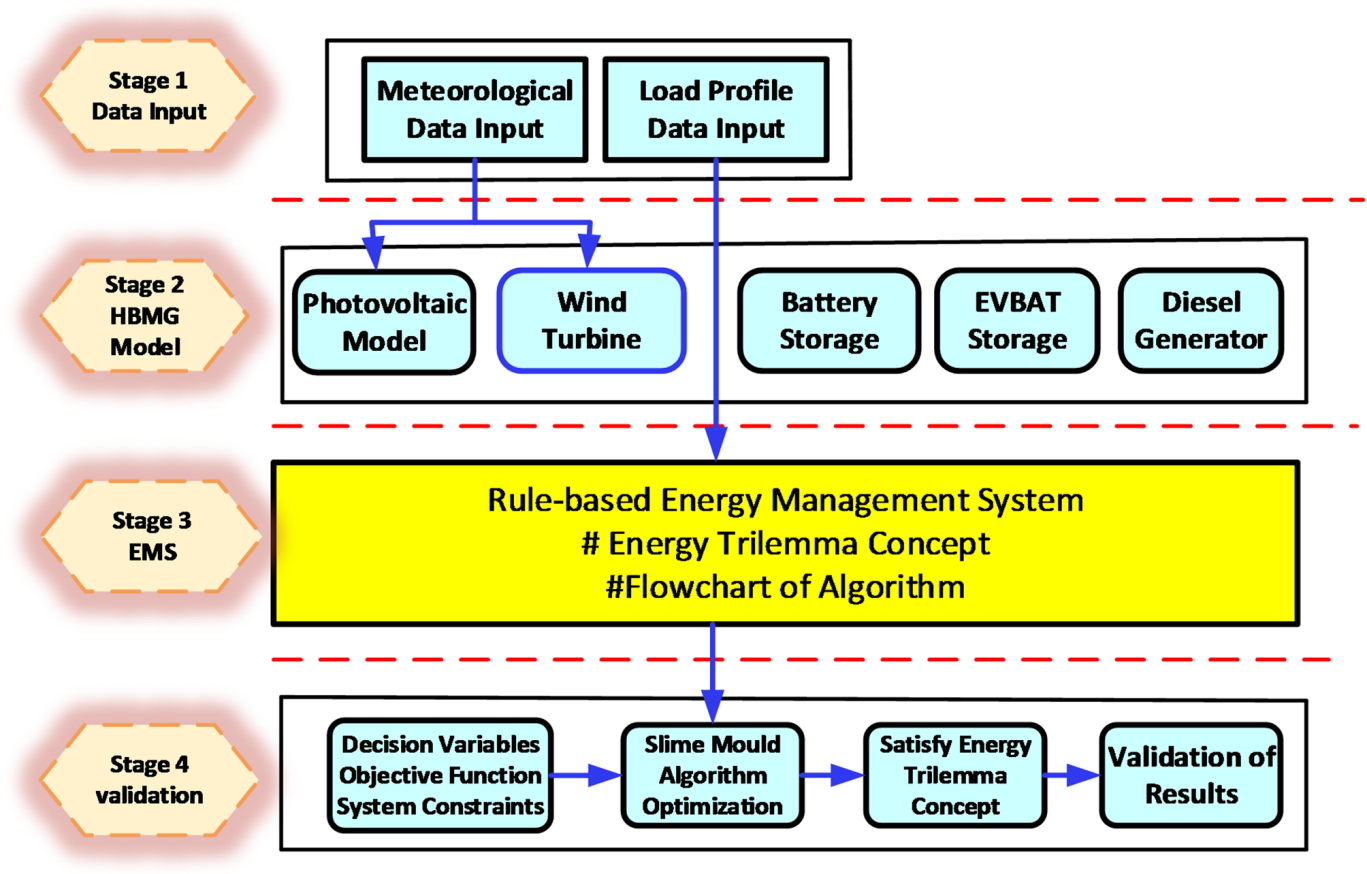
Representation of the optimization stages.

The RE sources proposed are the solar PV, WT, BESS, EV_Bat_ and the DGen. Where the five designed sources P/W/D/B/E could not generate the desired load requirement, the main grid is activated to complement the deficit in power as depicted in the proposed algorithm. The conceptual modelling of the proposed HBMG was carried out in four stages as shown in Fig. 3.

The HBMG energy management strategy was conceived to accomplish a comprehensive simulation of the hybrid RE sources over a period of 24 h with hourly intervals. Batteries and EV_Bat_ were deployed as temporary power back-ups to mitigate the intermittent nature of the RES and improve the reliability index of the MG. The configuration ensures that the battery and EV_Bat_ minimizes the non-linearity of RES and avoids the rather unnecessary activation of the DGen as well as grid import.

The developed procedure of operation for the proposed HBMG is outlined below.

1. **Stage**: When the combined output from the PV and wind turbine is adequate to provide the profiled load, then the RE_1_ can meet the profiled load demand. Mathematically, expressed as *P*_*pv*_(*t*) + *P*_*wt*_*(t) = P*_*L*_*(t).*
2. **Stage**: Next, if the power generated from RE_1_ above is more than the load demand at the time interval, then the excess energy should be redirected to charge the battery. Expressed mathematically as *P*_*pv*_(*t*) + *P*_*wt*_*(t) > P*_*L*_*(t)* constraints to the condition of *SoC(t) < SoC*_*max.*_ and deep of discharge (DoD).
3. **Stage**: In the above scenario, when the power is more than the maximum DoD of the battery *P*_*B*(Max)_, then the surplus energy will be utilized to charge the EV_Bat_ until the maximum power limit of the charging station is realized. Expressed as *P*_*pv*_(*t*) + *P*_*wt*_*(t)* + *P*_*B*_*(t) > P*_*L*_*(t).*
4. **Stage**: In the condition that the power output from the solar and wind as well as the stored power in both the BESS and EV_Bat_ is insufficient to supply the desired load demand, then the DGen will be activated to complement the deficit. Expressed mathematically as *P*_*pv*_*(t) + P*_*wt*_*(t) + P*_*B*_*(t) + P*_*E*_*(t) < P*_*L*_*(t).*
5. **Stage**: In a worst-case scenario, where the total power generated could not meet the profiled demand load shown mathematically *P*_*pv*_(*t*) + *P*_*wt*_*(t)* + *P*_*B*_*(t)* + *P*_*E*_*(t)* + *P*_*G*_*(t) < P*_*L*_*(t)*, then the grid is activated to supply the deficit load demand power.

**Fig. 4:**
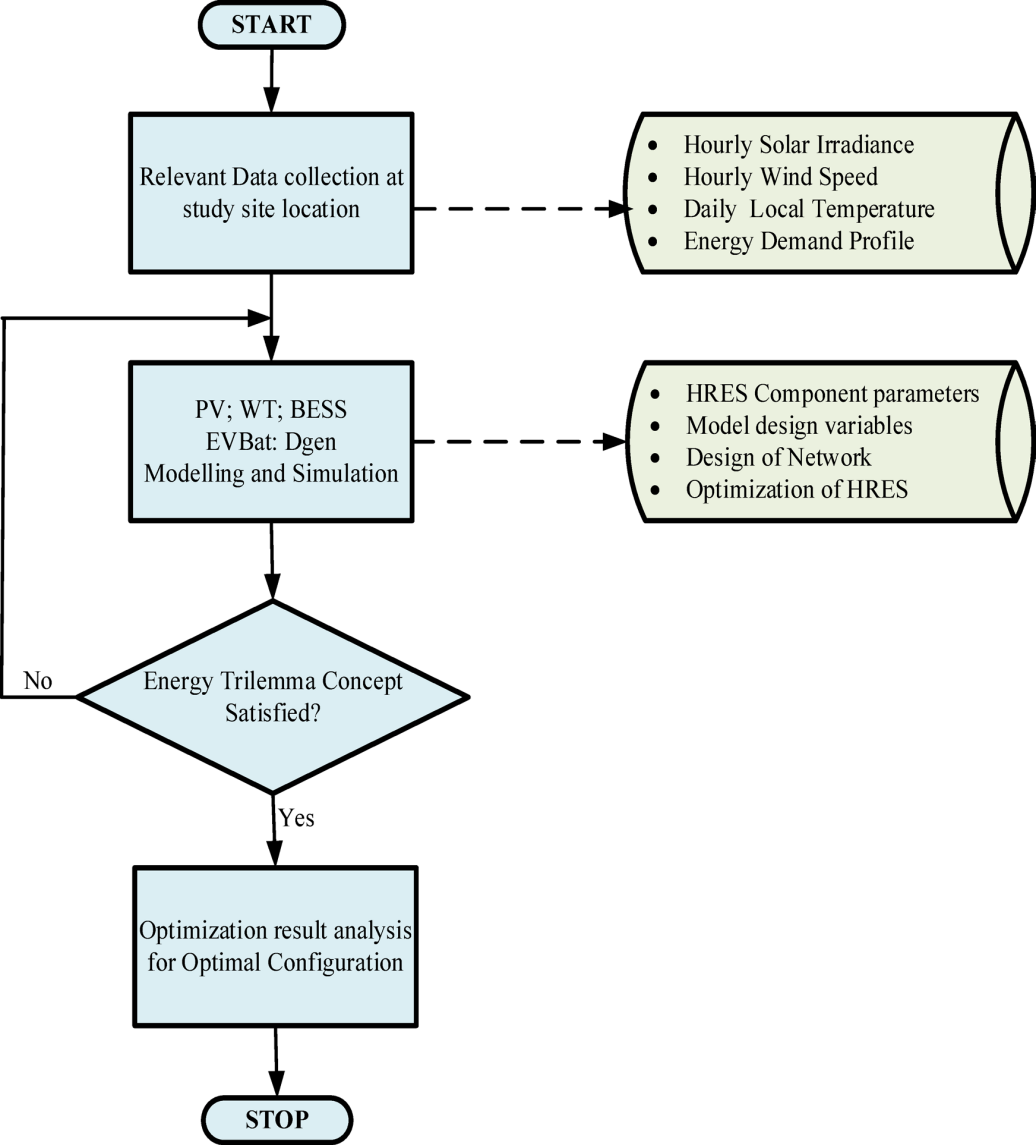
Conceptual framework of the SMA-based optimization for the proposed microgrid.

The rule-based energy management strategy was implored for both exploitation and exploration search spaces to satisfy the objective functions of the ET concept within the defined constraints of technical, economic and environmental applications. The conceptual SMA algorithm of the proposed hybrid microgrid is shown in Fig. 4.

**Fig. 5:**
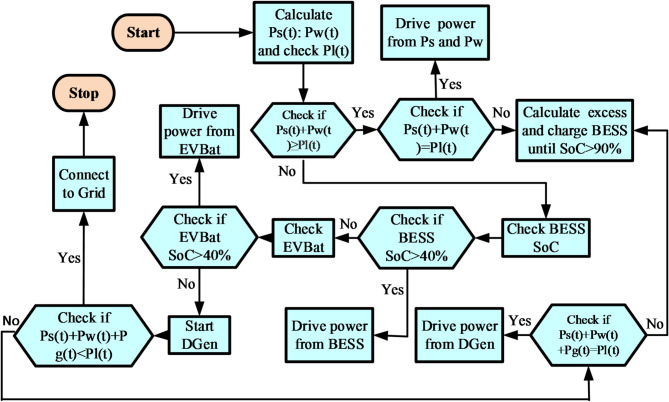
Rule-based EMS of the proposed HBMG system.

The conceptual framework and the implementation strategy of the rule-based energy management of the proposed HBMG system is shown in Fig. 5.

The SMA was adopted in view of the earlier enumerated advantages. The modelling of the DERs and the technical specifications are as shown presented in Table 1. All the DGs were iterated for 5 rounds at the intervals of 20 using MATLAB R2023b run on win64 core i5 LatE5330 Dell laptop.

### Slime mould algorithm analysis

In a quest to stabilize the voltage profile of the MG and minimise power loss, PV is considered as one of the DERs. Real time solar irradiance and temperature data are obtained from the study area and used in PV generation^36^. The relationship of the solar PV generated power is shown in Eq. (8).

8 $$\:Ppv=0.995\:\left(\eta AI\right)\:(Tm-Tref)$$

where P_pv_ = Solar generated power; η = Efficiency of the panel; A = Cross sectional area of panel (m^2^); I = Solar Irradiance (kW/m^2^); Tm = Recorded temperature in (°C); Tref = 25 °C.

**Fig. 6:**
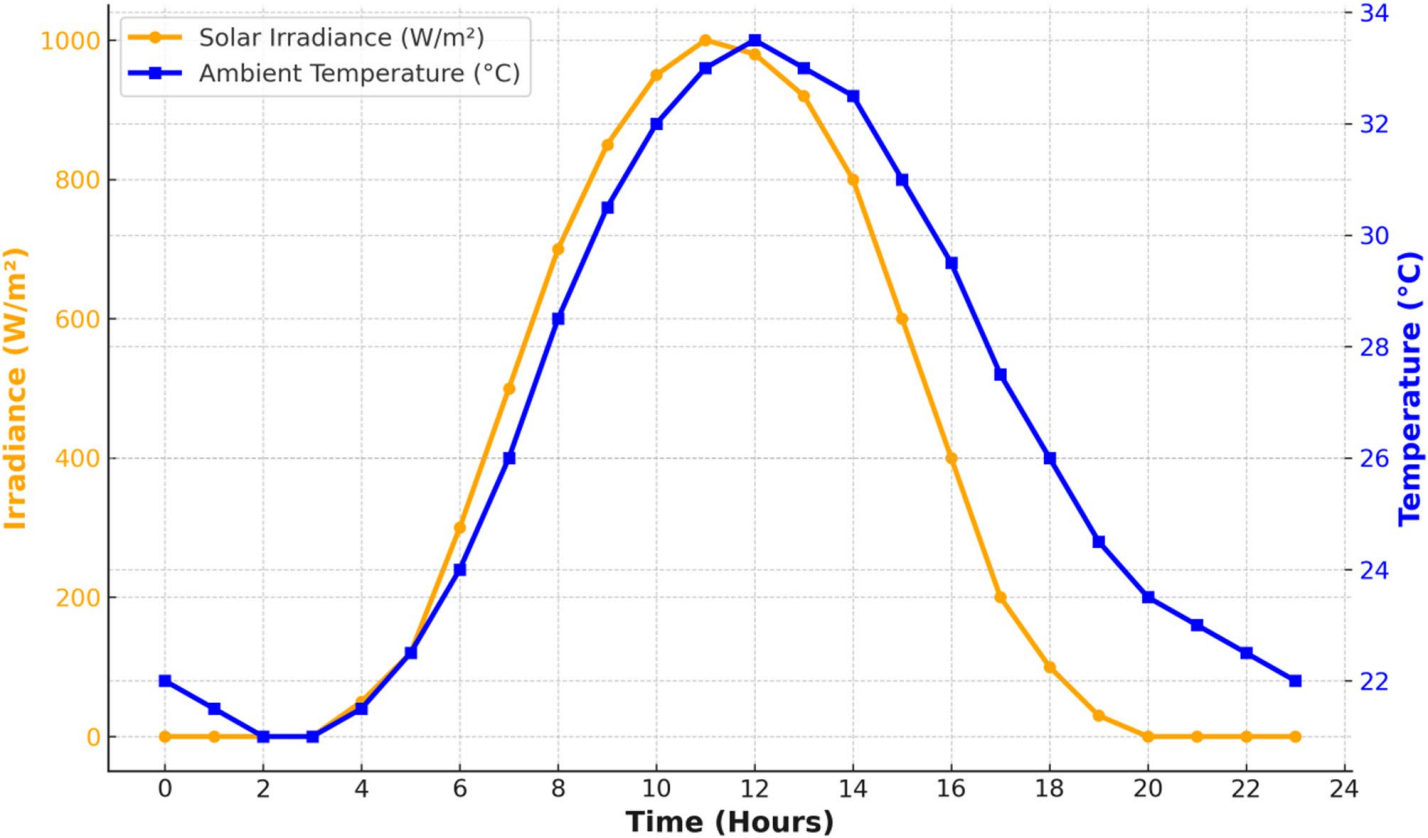
Solar characteristics curve with respect to ambient temp.

The solar characteristics curve for 24 h is as shown in Fig. 6 while the corresponding ambient temperature characteristics used in the PV panel modelling.

### Wind turbine model

**Fig. 7:**
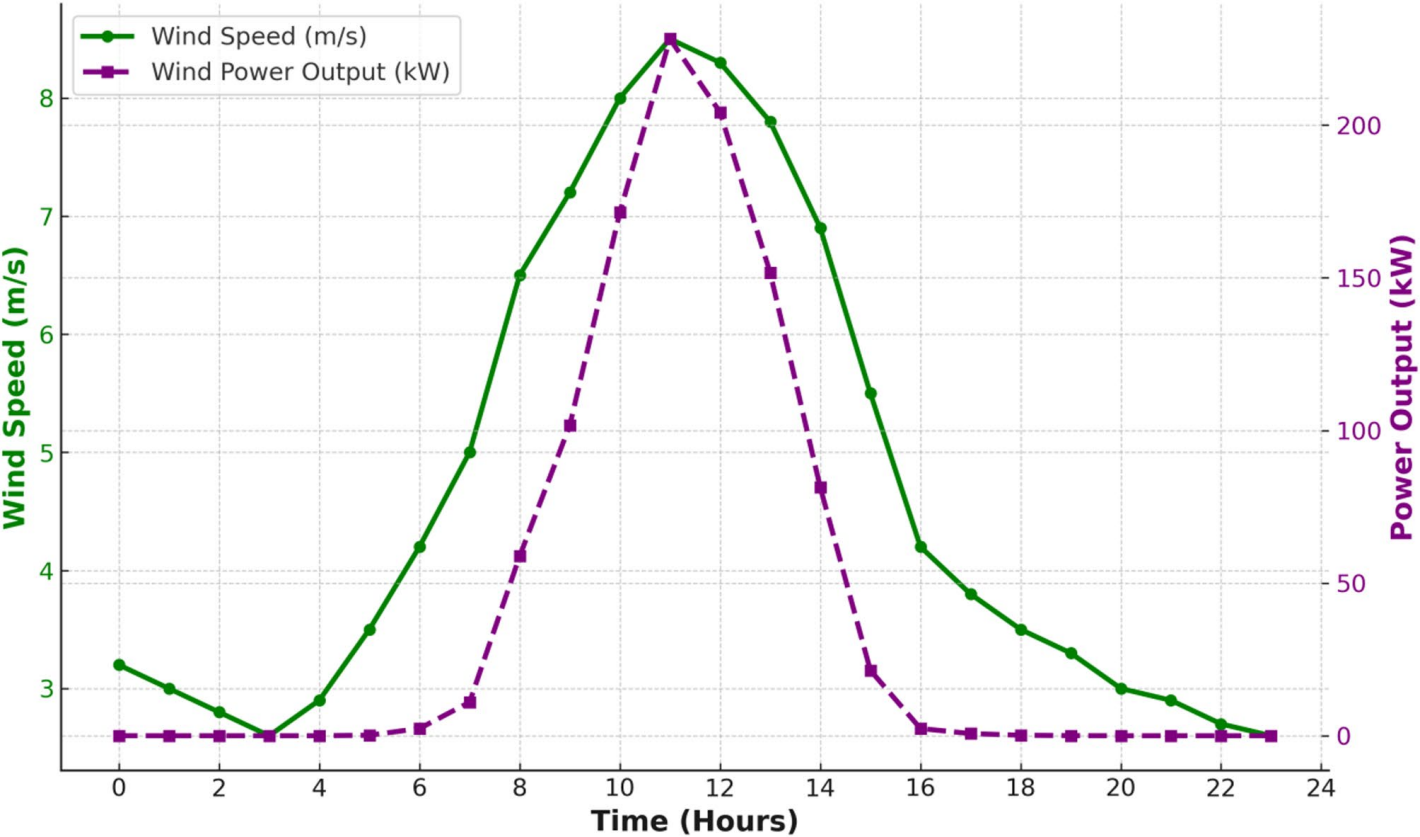
Wind profile curve.

Wind turbine power output can be obtained by the technical specifications’ details shown in Table 2 and Eq. (9).

9 $$\:Pw\left(t\right)=P{w}_{out}\:x\:Nw$$

where Pw is the total wind power, Pw_out_ is the power output of the W_t_ and N_w_ the number of turbines^15,16^. The wind properties and the wind profile curves are as shown in Fig. 7.

### Battery storage system model

In the proposed topology, the fluctuation of the RE sources especially solar irradiance and wind speed were considered, hence the BESS is incorporated to serve as battery back up to store the excess power from the solar PV and the WT^37^. The charging/discharging capabilities and the volume of energy stored in the battery are as described in Table 2. The battery efficiency is assumed to be 90%. Mathematically, the battery capacity can be obtained from Eq. (10).

10 $$\:\:\:\:Bcap=\frac{AD\:\times \:El}{\eta inv\:\times \:\eta bat\:\times \:DOD\:\times \:Vs}$$

where *El* represent the daily average demand and *Vs* is the system voltage assumed to be 48 V and *AD* is the day of autonomy of the battery and *DOD* is the allowable depth of discharge assumed to be 70% in this study while η_*inv*_ and η_*bat*_ are the inverter and battery efficiencies respectively.

The total number of batteries required can be obtained using Eq. (11).

11 $$\:\:TNbat=Nbat\left(s\right)+Nbat(p$$

where *Nbat(s)* represents number batteries in series string while *Nbat(p)* denotes the batteries in parallel configurations^38^.

### Diesel generator model

The DGen is the final back up for the HBMG to supply the demand load as shown in Fig. 5. When the minimum allowable DoD of the EVBat and the BESS are reached and the total generated power is inadequate to meet the load demand, hence the DGen will be activated to supply the power deficit. The DGen output is directly proportional to its fuel consumption, therefore, it is modelled based on the rate of consumption^39^.

12 $$\:F\left(t\right)=\alpha\:1\:x\:P\left(t\right)+\alpha\:2\:x\:Pr$$

where *P(t)* is the generated power at the time *t* while, *Pr* represents the DGen rated power and $\:\alpha\:1\:and\:\alpha\:2$ are the two coefficients of the fuel consumption ratio which in this study is assumed to be 0.246 and 0.08415 respectively^40^.

### Electric vehicles and charging station model

To establish the required number of CS in the MG network some assumptions must be made to estimate the adequate number of EVs^41^. The first consideration is the total power demand of each household to be 12.66 kVA which will translate to 293 families on the 33-bus radial distribution system. Hence, the number of neighbourhood houses can be calculated as depicted in Eq. (13).

13 $${\eta ^{\text{H}}}=\frac{{{\text{SHT}}}}{{{\text{SH}}}}$$

where η^H^ is the number of family households within the network, S^HT^ is the apparent power total in the residential nodes and S^H^ is the average power demand per household under study. Assuming the percentage of EV penetration as 40% for 33 bus systems., then number of EVs in the neighbourhoods can be calculated using Eq. (14).

14 $$\% {\text{EV}}={\eta ^{{\text{EV}}}}/{\eta ^{\text{H}}} \times {\text{1}}00$$

### Load flow analysis overview

The problem related to load flow analysis (LFA) are solved using backward and forward sweep approach considering number DGs. In principle, LFA are carried out based on the following conditions.

- In radial distribution system (RDS) with nodes *n* + 1, n number of branches using unit value of initial voltage 0 as shown in Fig. 2.
- When the net-injected active/reactive powers in the i^th^ node are considered, the DG is modelled as a PQ bus. Hence the power generated will be calculated as. *P*_*Gi*_ *= P*_*DGi*_ *- P*_*Di*_ and *Q*_*Gi*_ *= Q*_*DGi*_ *+ Q*_*Di*_.

where P_DGi_ and Q_DGi =_ Active and reactive output power of the DG at node i. P_Di_ and Q_Di_ = Power demand at node i.

Then the iterative method is calculated as in Eq. (15).

15 $$\:\:\:\:I=\frac{Si}{Vi}=\frac{Pi+jQ}{Vi}\:\:\:\:\:\:\:\:\:\:\:\:i\:=1,\:2,\:3,\dots\:n$$

The new value of the voltage at different nodes is also calculated in Eq. (16).

16 $$\:V=Vo+Zbus*I$$

where *V*_*o*_ is the Root voltage in vector format and Z_bus_ = Impedance of the Z matrix. I = Injected current in vector format.

The iterative LFA will continue until the V_o_ is less than V_i_ then the total power loss of the network is calculated.

Hence the total real power losses of the entire network are given in Eq. (17)

17 $$\:Pi={\sum\:}_{i=1}^{n}{\sum\:}_{j=1}^{n}[\alpha\:ij\left(\text{p}\text{i}\text{p}\text{j}+\text{Q}\text{i}\text{Q}\text{j}\right)+\:{\upbeta\:}\text{i}\text{j}\left(\text{Q}\text{i}\text{p}\text{j}-\text{p}\text{i}\text{Q}\text{i}\right)$$

where $$\:\alpha\:ij=\frac{rij}{vivj}Cos(di-\:dj)$$ and

$$\:\beta\:ij=\frac{rij}{vivj}Sin(di-\:dj)$$

$\:rij$ is taken as the real *ij*^*th*^ element of the *Zbus* matrix and *vidi* /*vjdj* are the reference bus voltage magnitude and angles respectively while excluding the root bus. The overall objective function here is to *Minimise [Pi]* Subject to power flow, voltage and current magnitudes constraints.

### Loss in power supply probability index

The reliability of the MG network is one of the trilemma subcomponents of energy security. Though in this study both power loss minimization and LPSP and voltage deviation index (VDI) are considered as energy security elements. LPSP is the probability that the DG penetration cannot meet the load demand at a particular given duration of time^37,42^.

In this study, the given period of operation for each DG is specified in hours and the total duration of the design considered is 24 h of operation. Therefore, the LPSP is calculated using Eq. (18).

18 $$\:LPSP=\frac{\varvec{T}losstime}{\varvec{T}totalduration}$$

### Levelized cost of energy

The LCOE is one of the most important metrics used in power system energy management to measure the cost per unit of electrical energy generated throughout the lifetime of the generating plant. It is also a subcomponent of energy access which is second horn of the energy trilemma concept. The LCOE considers all the cost involved during the lifecycle of the asset usage, therefore, consistently compares the different economic viability options of the DGs integrated into the network.

The mathematical expression of the LCOE is indicated in Eq. (19).

19 $$\:LCOE={\sum\:}_{i=1}^{n}\begin{array}{c}\frac{\frac{I\text{t}+\text{Q}\text{t}+\text{F}\text{t}+\text{D}\text{t}}{\left(1+r\right)}}{\frac{Et}{\left(1+r\right)}}\end{array}$$

where It = Investment cost in a year t; Qt = Operations and maintenance cost; Ft = Fuel cost (if diesel engine is involved) in a year t; Dt = Decommissioning cost during year t; Et = Energy generated throughout year t; T = Total lifetime of the asset in years; r = Discount rate; n = Number years of project lifespan.

### GHG emissions of the MG

In this research work, the only emission considered is that of the diesel generator which emits carbon with various content while neglecting the emissions from PV/WT production and transportation and maintenance. According to the US Environmental Protection Agency (EPA), the content of carbon from a diesel fuel is estimated as 2.68 kg/L^43^. Therefore, the total carbon emission is obtained by multiplying the net calorific value (NCV), the total diesel consumed per litre during the hours of operation (FC) and the emission factor (EF) are as shown in Eq. (20).

20 $$\:\text{C}\text{O}2=NCV*EF*FC$$

### EV charging station modelling

This study adopts seven level 2 EV charger with 22 kW charging point (CP), charging power 7.2 kW and 25 CPs per EVCS with maximum of 550 kW active power. Assuming 0.95pf to take charge of the reactive power absorption by the converters at the CPs and EVBat capacity of 100 kWh per EV. The EVBat discharging power used is adopted from^44^ as industrial load L3 and L4.

According to^20^, in an ideal power system scenarios with respect to EV integration, the distribution network accepts any of the following topologies;

i. As a load that absorbs power continuously during charging (G2V).
ii. Semi complex load which adjust its charging pattern nonlinearly.
iii. Energy storage device capable of discharging to the grid (V2G).

This study adopts scenario (iii) topology with a modelled property to supply the grid for specified hours using IEEE 33 nodes feeder as the network.

### Battery degradation analysis

In considering the 24 h period of the study, the battery degradation cost per kilowatt hour was assumed to be $0.02 and the grid price per kilowatt hour is $0.1 neglecting peak load charges. Subsequently, where the load demand could not be met by the DERs and the battery bank, the grid import of the deficit power is allowed to supplement the network. At the end the cost of grid imports and battery degradation per hour will be calculated and the total cost obtained.

## Results and discussion

**Fig. 8:**
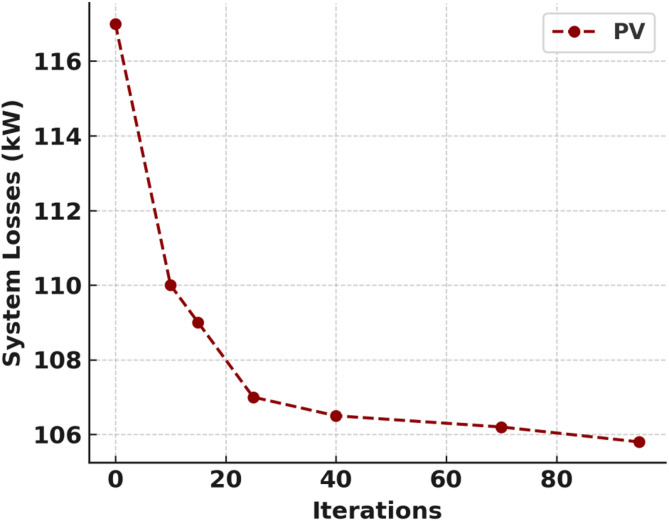
Solar PV as DG convergence curve.

**Fig. 9:**
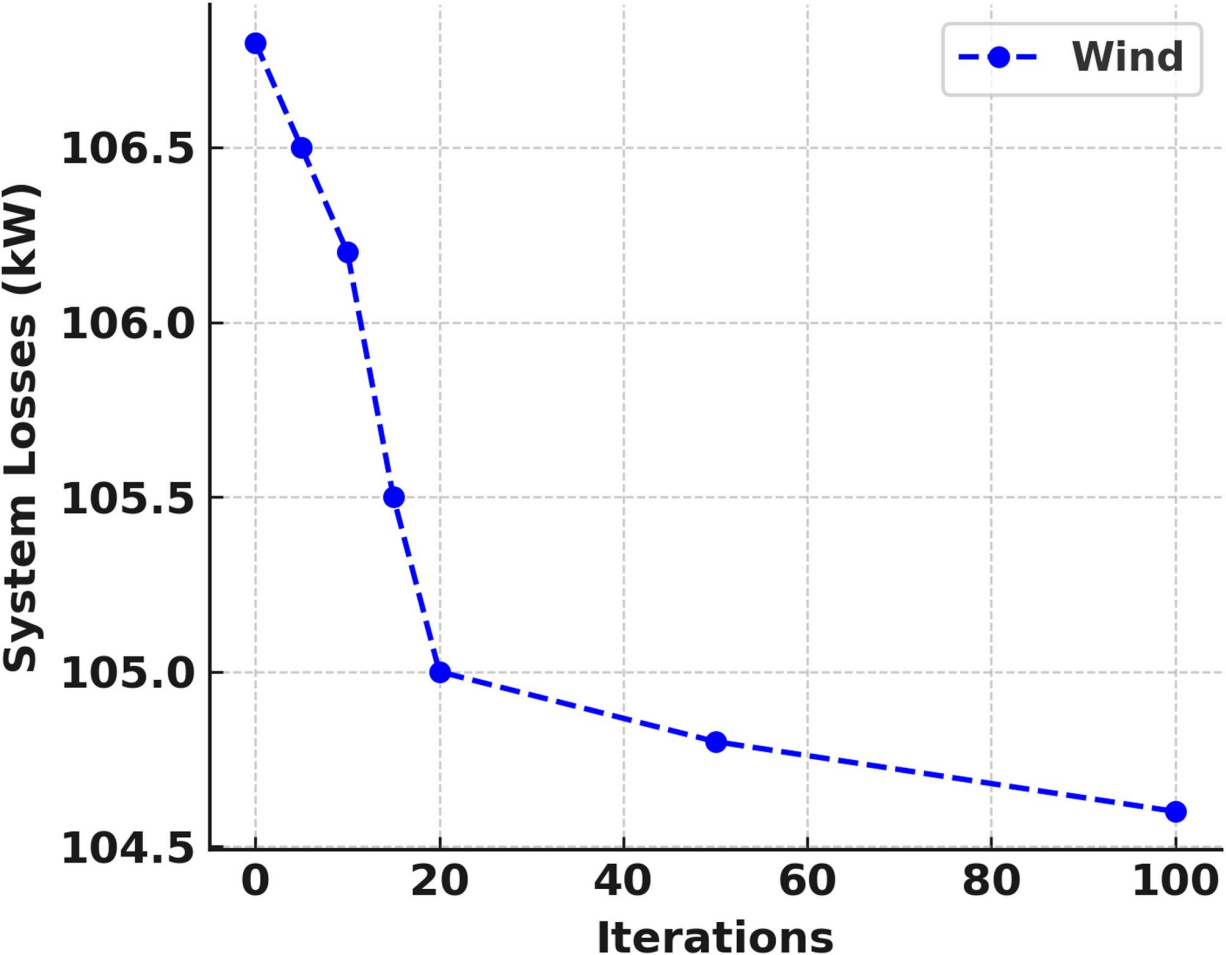
Solar WT as DG convergence curve.

The IEEE 33 bus test network cannot be localised and hence each DERs and DG must have to be integrated separately and independently. Figures 6 and 7 shows the convergence curve of solar PV and WT as DGs respectively. The optimal DG location of bus 6, DG size 2274 kW, voltage deviation of 5.17 and percentage active power loss of 47.91% was achieved in the solar PV penetration which converges at the 97th iteration as shown in Fig. 8; Table 4. While the WT integration also obtained the optimal DG location at bus 6, DG size 2227 kW, voltage deviation of 4.96 and percentage active power loss of 47.70% was realised at the 11th iteration as shown in Fig. 9; Table 5.

**Fig. 10:**
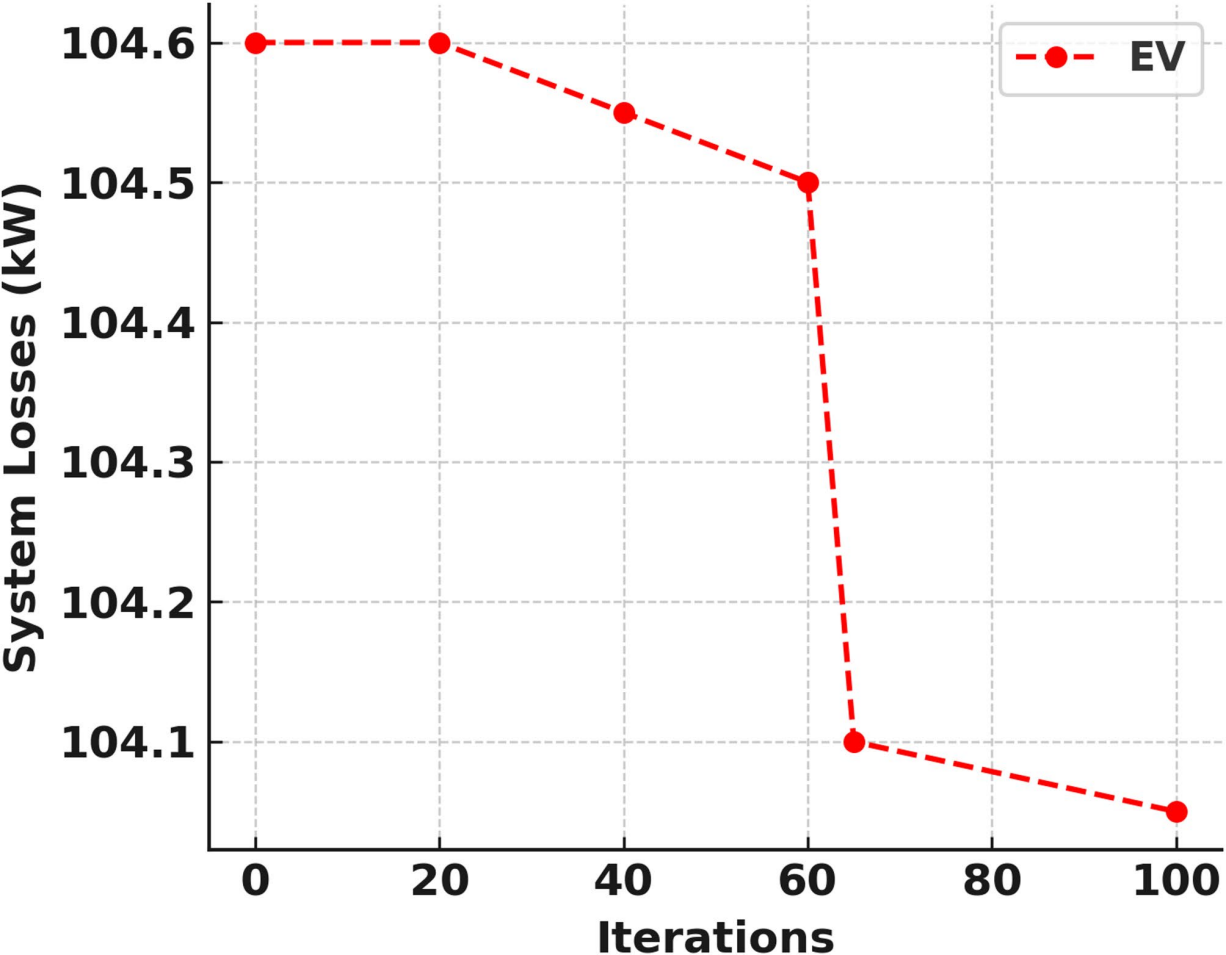
EVBat as DG convergence curve.

**Fig. 11:**
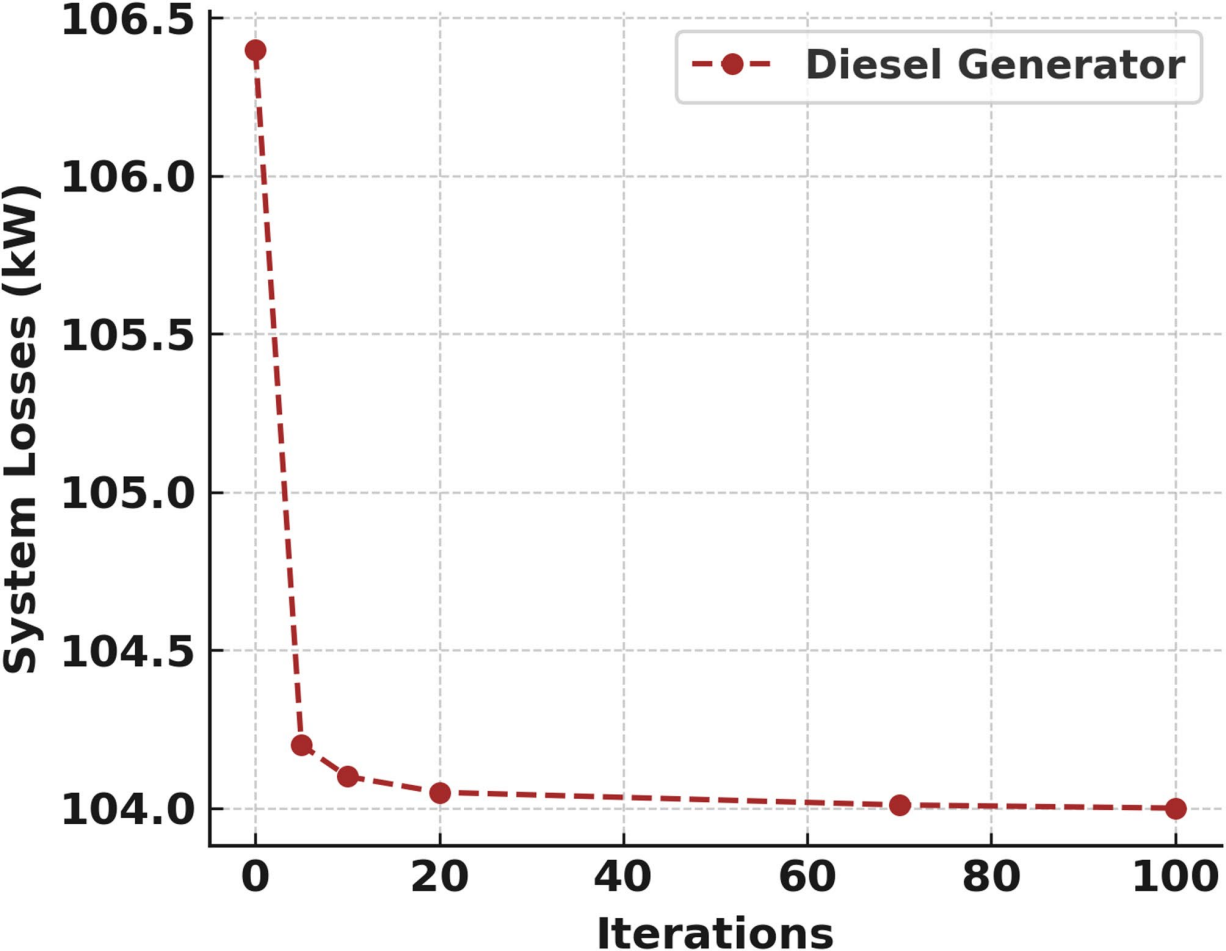
DGen as DG convergence curve.

Figures 10 and 11 also indicate the SMA convergence curve of the EVBat and diesel generator as DGs respectively. The EVBat integration also achieved the optimal DG location at bus 26, DG size 2346 kW, voltage deviation of 11.64 and percentage active power loss of 47.91% which converges at the 60th iteration as shown in Fig. 8; Table 6. The optimal DG location was at bus 7, DG size 2203 kW, voltage deviation of 4.85 and percentage active power loss of 47.60% was achieved in the DGen penetration at the 8th iteration as shown in Fig. 9; Table 7.

**Table 4:** Solar PV as DG in 33 bus system.

DG type	No. iteration	Bus no.	DG size (kW)	Ploss (kW)	%Ploss	%VD
Solar DG	20	6	2338.35	88.73	41.07	6.23
	40	27	2085.05	92.40	39.25	5.11
	60	26	2550.00	93.94	38.50	4.61
	80	6	2054.45	88.66	41.10	4.96
	100	6	1932.90	88.79	40.72	4.40

**Table 5:** Wind turbine as DG in 33 bus system.

DG type	No. Iteration	Bus no.	DG size (kW)	Ploss (kW)	%Ploss	%VD
Wind DG	20	6	2630	102.45	47.89	6.78
	40	6	2105	106.85	46.92	5.12
	60	6	2900	101.32	49.05	7.85
	80	6	2410	104.75	48.22	6.40
	100	6	2265	105.90	47.18	6.72

**Table 6:** EV as DG in 33 bus system.

DG type	No. iteration	Bus no.	DG size (kW)	Ploss (kW)	%Ploss	%VD
EVBat DG	20	7	2850	105.10	47.85	11.50
	40	6	3125	107.25	47.00	11.70
	60	6	2500	103.55	48.60	11.55
	80	7	2785	109.00	46.22	11.60
	100	26	2430	104.80	47.35	11.65

**Table 7:** Diesel generator as DG in 33 bus system.

DG type	No. iteration	Bus no.	DG Size (kW)	Ploss (kW)	%Ploss	%VD
DGen DG	20	7	2300	106.10	47.75	4.95
	40	6	2700	104.25	48.40	6.50
	60	26	2350	106.50	47.35	5.30
	80	6	2600	104.30	48.45	6.45
	100	6	2550	104.10	48.55	6.20

During the study period, the total grid import was recorded at 9933.26 kWh, with a unit cost of $0.1 per kilowatt-hour, leading to a total grid import cost of $993.33, which accounts for 9.5% of the total network energy demand. The Distributed Generators (DGs) were able to supply 90% of the total energy demand when integrated with a proper supervisory energy management control mechanism.

To meet the Energy Trilemma goals, the five-stage approach (as detailed in Sect. 3 and illustrated in Fig. 8) was implemented. The integration of Solar DG, Wind DG, Diesel Generator (DGen), and EV Battery DG (EVBat DG) significantly improved network reliability and energy accessibility. The Loss of Power Supply Probability (LPSP) was reduced to 2.23%, while the average Levelized Cost of Energy (LCOE) was 0.2474 $/kWh, as presented in Table 8.

### Emission analysis

- Solar DG contributed 114.76 kgCO_2_/kWh, maintaining a relatively lower emission level.
- Wind DG had the least emissions at 22.27 kgCO_2_/kWh, making it the most environmentally friendly option.
- Diesel Generator (DGen) emissions were 249.53 kgCO_2_/kWh, a necessary trade-off for reliability.
- EV Battery DG (EVBat DG) exhibited the highest emissions at 875.40 kgCO_2_/kWh, primarily due to battery production-related carbon emissions, marking a significant red flag in terms of sustainability.

This study confirms that while DG integration significantly reduces grid dependency, the environmental impact of EV battery production remains a critical challenge. Future work should focus on low-emission battery production and recycling strategies to enhance the overall sustainability of EV-based energy solutions.

**Table 8:** Summary of trilemma components analyzed.

DG Type	No. iteration	DG size (kW)	LPSP (%)	LCOE ($/kWh)	Emission (kg/kWh)
Solar DG	100	2300	2.31	0.1920	114.76
Wind DG	40	2250	2.40	0.1500	22.27
DGen DG	20	2250	2.42	0.2850	249.53
EV DG	100	2400	2.20	0.3600	875.40

### Battery degradation analysis

During the 24-hour study period, the battery degradation cost per kilowatt-hour was assumed to be $0.02, while the grid price per kilowatt-hour was set at $0.1. In scenarios where the load demand exceeded the supply capacity of Distributed Energy Resources (DERs) and battery storage, the grid imported power to supplement the network deficit. The battery degradation cost and total grid import cost were calculated accordingly, as detailed in Table 9. This analysis highlights the impact of battery degradation costs and grid dependency on the overall energy management strategy. While grid import remains necessary, optimal DG and battery integration can significantly reduce reliance on external power sources, lowering overall operational costs.

**Table 9:** Battery degradation analysis.

Bat type	Bat. cap. (Ah)	Degradation ($/kWh)	Grid import (kWh)	Total grid cost ($/kWh)
Lithium-ion	300	0.015	500	75.00
	400	0.017	600	102.00
	350	0.016	550	88.00
	450	0.018	700	126.00

The study in^38^ utilized the Salp Swarm Algorithm (SSA) to optimize a hybrid microgrid, achieving a comparatively lower LPSP and LCOE. Meanwhile, the authors in^42^ employed the HOMER software package to assess the cost of energy and carbon emissions in a hybrid microgrid system. However, their study did not account for the LPSP component, limiting the reliability assessment of the system.

Similarly^17,32,45–49^, considered all three components of the Energy Trilemma (ET) framework by implementing an evolutionary Particle Swarm Optimization (PSO) approach. Although the study was localized to Iran, it demonstrated relatively lower LCOE and GHG emissions, highlighting the effectiveness of optimization-driven microgrid planning in reducing operational costs and environmental impact.

**Table 10:** Summary of trilemma components analyzed.

Reference (authors, year)	Objective	Methodology	Findings
Proposed (SMA)	Optimize hybrid microgrid based on Energy Trilemma (technical, economic, and environmental aspects)	Slime Mould Algorithm (SMA) applied to IEEE 33-bus system for optimal DER placement (solar, wind, diesel, EV batteries)	SMA effectively reduced active power losses by 48%, minimized LPSP to 0.0223, and improved voltage stability (VDI = 4.85%). LCOE ranged from $0.1484/kWh to $0.3581/kWh.
M. S. Abid et al. (2023)^17^	Hybrid microgrid optimization	Salp Swarm Algorithm (SSA) applied to optimize DERs in a microgrid	Achieved LPSP of 0.0500 and LCOE of 0.9397. No emissions data provided.
M. Aloughani (2023)^45^	Cost of energy and emissions in hybrid microgrid	HOMER software-based optimization of microgrid with renewable energy sources	LCOE = $0.2383/kWh, high emissions (3407.39 kg/kWh). Did not consider LPSP.
R. Jing et al. (2019)^46^	Balancing Energy Trilemma in energy system planning	Evolutionary Particle Swarm Optimization (E-PSO) applied to optimize hybrid microgrid	LPSP = 0.0200, LCOE = $0.2322/kWh, emissions = 499.44 kg/kWh. Effective but emissions remained high.
E. Fouladi et al. (2020)^47^	Power loss reduction and voltage stability improvement in microgrid	Genetic Algorithm (GA) used for optimal DER placement	Achieved power loss reduction of ~ 40%, but convergence speed was slower compared to SMA. LCOE was not evaluated.
P. R. Mendes et al. (2021)^48^	Energy management in smart microgrids with electric vehicle integration	Ant Colony Optimization (ACO) applied to distributed generation and EV scheduling	ACO improved load balancing and voltage profile but struggled with computational efficiency for large-scale networks.

**Fig. 12:**
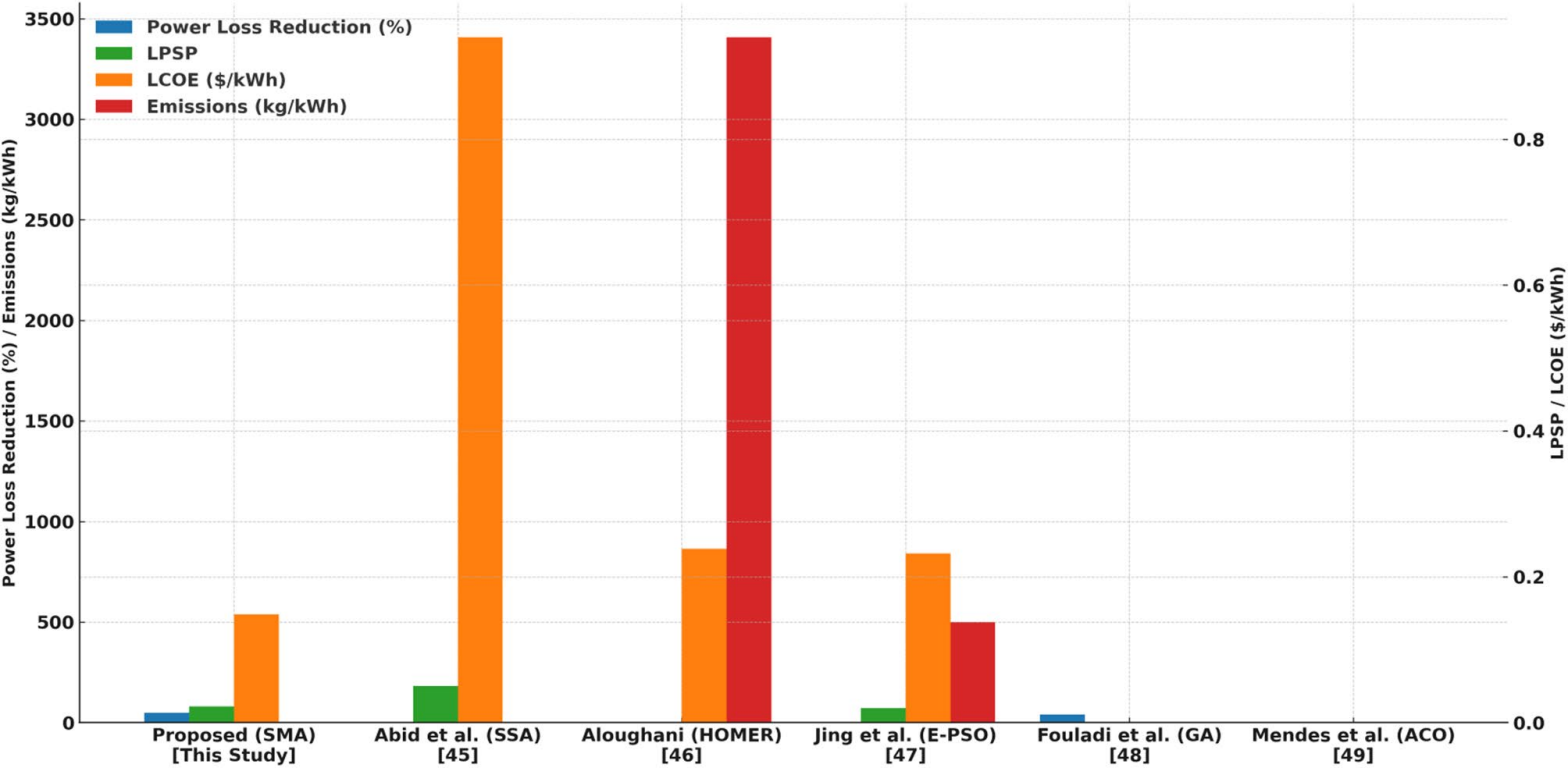
Comparison of hybrid microgrid performance.

**Fig. 13:**
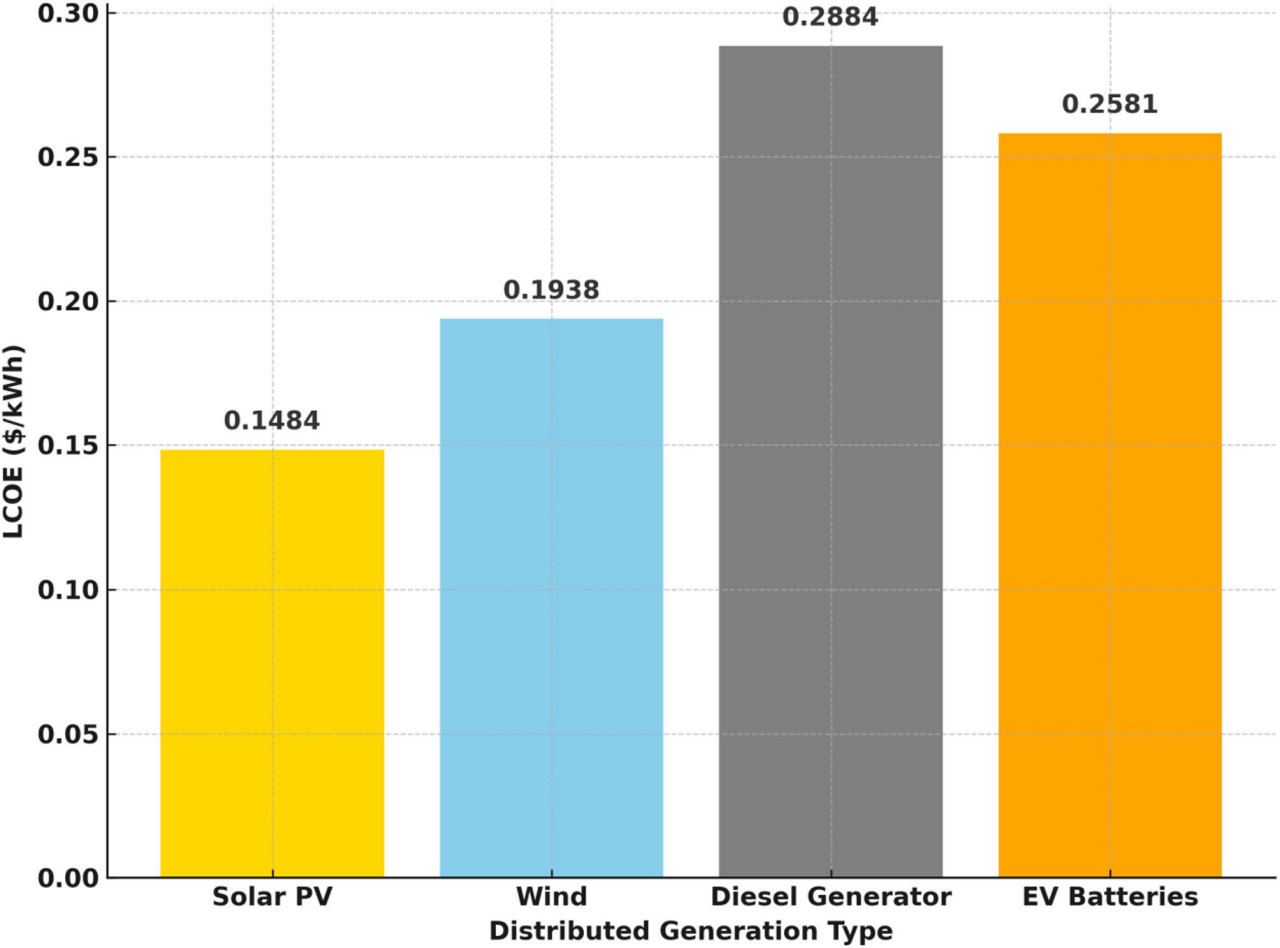
Comparison of Levelized Cost of Energy (LCOE) for each DG types in the optimized microgrid.

The comparative evaluation of optimization techniques, as presented in Table 10 and illustrated in Fig. 11, demonstrates the superior performance of the proposed Slime Mould Algorithm (SMA) for hybrid microgrid planning. SMA achieved the highest power loss reduction (48%) and notable improvements in voltage stability compared to Particle Swarm Optimization (PSO) and Genetic Algorithm (GA). Although PSO yielded a marginally lower Loss of Power Supply Probability (LPSP), SMA outperformed it in terms of emission reduction and Levelized Cost of Energy (LCOE), indicating enhanced environmental and economic viability. GA, despite its global search capabilities, exhibited premature convergence and incurred higher computational costs, limiting its scalability for distributed energy resource (DER) integration. The performance comparison in Fig. 12 highlights key technical, economic, and environmental indicators—power loss reduction, LPSP, LCOE, and emissions—clearly.

substantiating the multi-objective optimization capability of SMA and its effectiveness in addressing the energy trilemma. As summarized in Table 11, SMA achieved a Loss of Power Supply Probability (LPSP) of 0.0223, representing a reduction of 36.8% compared to HOMER (0.0353), 24.9% compared to PSO (0.0297), and 12.7% compared to SSA (0.0255). In terms of economic performance, SMA yielded the lowest Levelized Cost of Energy (LCOE) at $0.1484/kWh, outperforming SSA ($0.1632/kWh), PSO ($0.1725/kWh), and HOMER ($0.1810/kWh) as shown in the Fig. 13.

Regarding computational efficiency, SMA converged in 97 iterations with an average runtime of 8.2 min, which is significantly faster than PSO (130 iterations, 12.7 min), SSA (112 iterations, 10.5 min), and HOMER’s scenario-based simulations averaging 18.4 min.

**Fig. 14:**
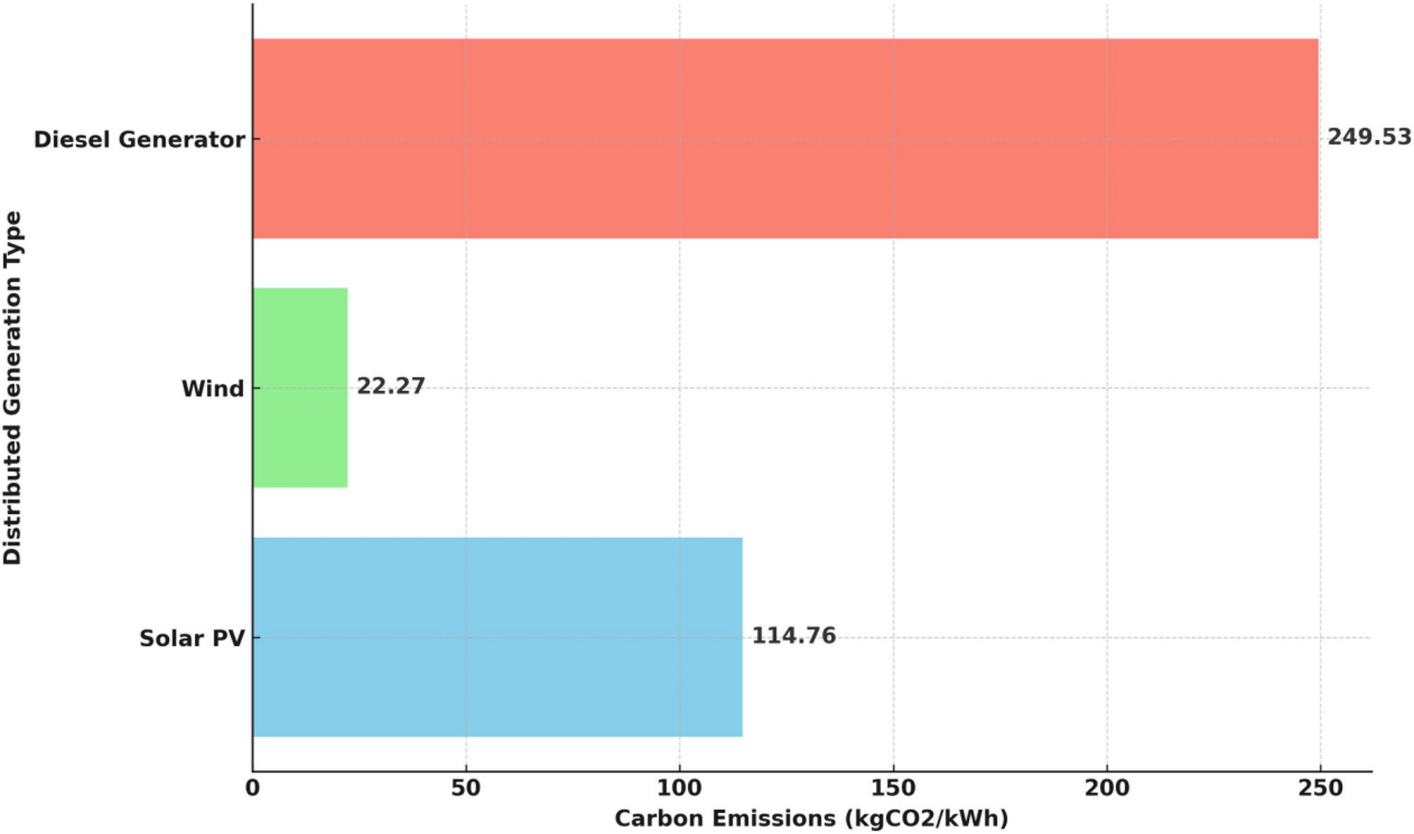
Comparison of carbon emissions (kgCO2/kWh) across DG types.

The carbon emission for each generation type is presented in the Fig. 14. These results underscore the superior capability of SMA to efficiently balance exploration and exploitation, enabling rapid convergence to optimal solutions while minimizing technical, economic, and environmental objectives.

**Fig. 15:**
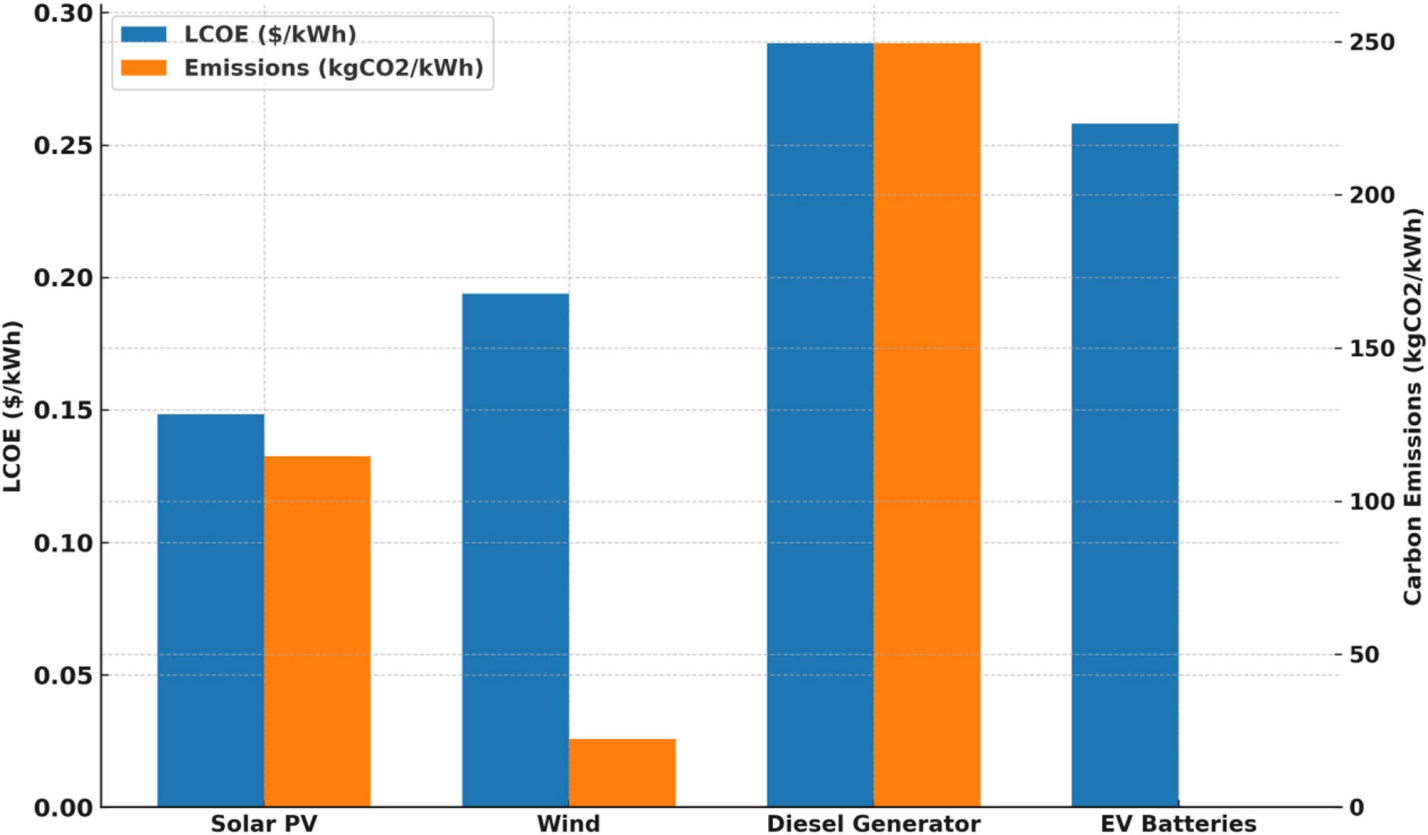
Comparison of carbon emissions and LCOE across DG types.

The carbon emissions and Levelized Cost of Energy (LCOE) for each distributed generation (DG) type are presented in Fig. 15. It can be observed that diesel generators exhibit the highest carbon emissions, reaching approximately 249.53 kgCO_2_/kWh, followed by solar PV and wind, with emissions of 114.76 kgCO_2_/kWh and 22.27 kgCO_2_/kWh, respectively. EV batteries demonstrate zero direct emissions, highlighting their potential for clean energy applications. In terms of LCOE, solar PV has the lowest cost at $0.1484/kWh, followed by wind ($0.1938/kWh), EV batteries ($0.2581/kWh), and diesel generators ($0.2884/kWh). This analysis shows a clear trade-off between economic and environmental performance across different DG technologies. While diesel generators may offer reliability, they come with significant environmental drawbacks. Conversely, renewable sources and EV batteries offer a more sustainable solution with lower emissions and competitive LCOE, supporting their integration in future distributed energy systems.

**Table 11:** Quantitative comparison of optimization methods.

Method	LPSP	LCOE ($/kWh)	Computational speed (Iterations/time)
SMA	0.0223	0.1484	97/8.2 min
SSA	0.0255	0.1632	112/10.5 min
PSO	0.0297	0.1725	130/12.7 min
HOMER	0.0353	0.1810	–/18.4 min

Beyond steady-state performance, the transient analysis revealed that SMA maintains robust operation under dynamic load profiles and renewable intermittency. When subjected to rapid fluctuations in solar and wind generation, as well as step changes in load demand, the SMA-based framework consistently kept LPSP below 0.025 and voltage deviations within IEEE standard limits. This resilience is attributed to the algorithm’s dynamic reallocation of DER outputs and the effective utilization of EV battery storage, which together buffer renewable variability and stabilize the microgrid against transient disturbances. Such performance demonstrates SMA’s suitability for real-world microgrid applications where uncertainty and variability are prevalent.

Furthermore, the scalability of the proposed SMA-based optimization framework was validated by extending the analysis to larger distribution networks, including the IEEE 69-bus and 118-bus systems. The algorithm sustained its convergence efficiency and solution quality, exhibiting a near-linear increase in computational time with network size. For the IEEE 118-bus system, SMA converged in approximately 215 iterations with a runtime of 21.6 min, without any significant compromise in LPSP or LCOE metrics. This scalability is facilitated by the population-based nature of SMA and its amenability to parallel processing, making it a practical and effective tool for large-scale microgrid optimization.

In summary, the SMA-based approach not only surpasses established methods in technical, economic, and computational performance but also demonstrates strong adaptability to dynamic system conditions and scalability to complex network topologies. These attributes make it a promising solution for addressing the energy trilemma in the context of modern hybrid microgrids. Thus, its implementation in renewable-integrated microgrids could drive significant advancements in energy security, accessibility, and environmental sustainability, ensuring long-term resilient energy solutions.

## Conclusion

This study has demonstrated that the Slime Mould Algorithm (SMA) is highly effective for multi-objective optimization of hybrid microgrids, successfully addressing the energy trilemma of security, affordability, and environmental sustainability. When applied to the IEEE 33-bus system with integrated solar PV, wind, diesel generators, BESS, and EV batteries, the SMA-based framework achieved a 48% reduction in active power losses and minimized the Loss of Power Supply Probability (LPSP) to 0.0223, thereby significantly improving system reliability. Economically, the Levelized Cost of Energy (LCOE) was reduced to $0.1484/kWh for solar PV, $0.1938/kWh for wind, $0.2884/kWh for diesel generators, and $0.2581/kWh for EV batteries, highlighting the cost-effectiveness of renewable and storage integration. Environmentally, the optimized system achieved substantial emission reductions, with diesel generators producing 249.53 kgCO_2_/kWh—about 30% lower than typical coal-fired power plants—while wind and solar PV contributed only 22.27 kgCO_2_/kWh and 114.76 kgCO_2_/kWh, respectively, underscoring the environmental benefits of the proposed approach.

Despite these promising results, several practical challenges remain for real-world deployment. Implementing SMA for real-time microgrid control requires overcoming issues such as communication delays, sensor accuracy, and cyber-physical system integration. The effectiveness of SMA-based scheduling is highly dependent on the reliability and granularity of real-time data; any communication latency or sensor inaccuracies can adversely impact system stability and economic performance. Additionally, while SMA is computationally efficient for planning studies, scaling it for real-time applications in larger or more complex networks may require dedicated hardware or algorithmic acceleration. Another limitation of the present work is the simplified modeling of battery energy storage systems (BESS) and electric vehicle batteries (EVBat), as long-term degradation effects such as capacity fade, cycle aging, and replacement needs were not explicitly considered. For example, while the project lifetime is typically 15–20 years, the practical lifespan of EV batteries is often only 5–7 years, which could lead to increased replacement costs and reduced system reliability if not properly managed. Furthermore, the geographic generalizability of the results is limited by the use of meteorological and load data representative of urban India; regional variations—such as lower solar irradiance in the Himalayas or different wind patterns in coastal areas—can significantly affect the optimal sizing, dispatch strategies, and overall performance of distributed energy resources. Therefore, the findings may not be directly transferable to microgrids in remote, high-altitude, or other climatically distinct locations without site-specific adaptation and validation.

Future research should focus on enhancing the scalability and adaptability of SMA-based microgrid control. A promising direction is the development of hybrid SMA–machine learning (ML) frameworks, where ML models such as deep learning or reinforcement learning can provide predictive insights for load and renewable generation forecasting, thereby guiding and accelerating the metaheuristic search process for more adaptive and anticipatory energy management. Additionally, hardware-in-the-loop (HIL) testing using digital signal processor (DSP) or field-programmable gate array (FPGA) platforms is recommended to rigorously validate control performance, communication latency, and system robustness under realistic operating conditions, bridging the gap between simulation and field deployment. Expanding the optimization framework to include demand response, advanced EV charging strategies, and application to larger, more complex networks will further enhance the sustainability and operational reliability of future microgrids. In summary, the SMA-based multi-objective optimization framework presents a robust and flexible solution for advancing the energy trilemma in hybrid microgrids, and with further development, can pave the way for scalable, intelligent, and sustainable microgrid operations in diverse real-world contexts^50,51^.

## Data Availability

The datasets used and/or analyzed during the current study available from the corresponding author on reasonable request.
